# ASO Author Reflections: The Heterogenicity of EGFR Mutations in Patients with Pulmonary Ground-Glass Opacities

**DOI:** 10.1245/s10434-025-16879-9

**Published:** 2025-01-20

**Authors:** Wen-Fang Tang, Wei-Zhao Huang, Wen-Zhao Zhong, Yi Liang

**Affiliations:** 1 Department of Cardiothoracic Surgery, Zhongshan City People’s Hospital, Zhongshan, Guangdong People’s Republic of China; 2 Guangdong Lung Cancer Institute, Guangdong Provincial People’s Hospital (Guangdong Academy of Medical Sciences), Southern Medical University, Guangzhou, Guangdong People’s Republic of China

## PAST

Non-small cell lung cancer (NSCLC) is considered to be a heterogeneous disease.^1^ Lung ground-glass opacities (GGOs), which are classified as an indolent subtype,^2^ have been increasingly detected owing to the popularity of low-dose computed tomography (LDCT). GGOs are more likely to be found in younger patients and female nonsmokers,^3^ and epidermal growth factor receptors (EGFR) are the most common therapeutic target in NSCLC.^4^ However, as it is the group in the earliest stage of lung cancer development, the correlations between EGFR mutation rates, age, and tumor characteristics of patients with GGOs remain unclear.

## PRESENT

Using the data of 1473 patients with GGOs from two cancer centers,^5^ the EGFR mutation rates were compared, stratified by different groups. In total, we found that those diagnosed at an age older than 50 years had a 47.0% increased likelihood of harboring EGFR mutations. Compared with the older group, the increased chance of harboring EGFR mutations for the patients with larger tumors, mixed GGOs, and IAC was greater in the younger group. Multivariate logistic regression indicated that age, GGOs types, tumor diameter and pathological types were significant predictors for EGFR mutations status. Furthermore, the distributions of EGFR subtypes varied in different age and GGOs diameter groups.

## FUTURE

The current study^5^ offered new insights into the tumor biology and treatment strategies of GGOs. To the authors’ knowledge, few large-scale studies have clearly explored the correlations between tumor features, age, and EGFR mutation rates in patients with GGOs. However, this study only included Asian patients, and did not include Western ethnicities. Most patients were only tested for EGFR mutations and lack comprehensive genotyping. A study including Asian and Western patients with comprehensive genetic testing is needed to further explore these differences.

## References

[1] Chen Z, Fillmore CM, Hammerman PS, et al. Non-small-cell lung cancers: a heterogeneous set of diseases. *Nat Rev Cancer*. 2014;14(8):535–46. 25056707 10.1038/nrc3775 PMC5712844

[2] Saji H, Okada M, Tsuboi M, et al. Segmentectomy versus lobectomy in small-sized peripheral non-small-cell lung cancer (JCOG0802/WJOG4607L): a multicentre, open-label, phase 3, randomised, controlled, non-inferiority trial. *Lancet*. 2022;399(10335):1607–17. 35461558 10.1016/S0140-6736(21)02333-3

[3] Ye T, Deng L, Wang S, et al. Lung adenocarcinomas manifesting as radiological part-solid nodules define a special clinical subtype. *J Thorac Oncol*. 2019;14(4):617–27. 30659988 10.1016/j.jtho.2018.12.030

[4] Shigematsu H, Lin L, Takahashi T, et al. Clinical and biological features associated with epidermal growth factor receptor gene mutations in lung cancers. *J Natl Cancer Inst*. 2005;97(5):339–46. 15741570 10.1093/jnci/dji055

[5] Tang W, Qiu Z, Chu X, et al. EGFR mutation rates correlate with age at diagnosis and tumor characteristics in patients with pulmonary ground-glass opacities. *Ann Surg Oncol*. 2024. 10.1245/s10434-024-16730-7. 39722087 10.1245/s10434-024-16730-7 PMC12130132

